# Identification and antitumor activity of a novel inhibitor of the NIMA-related kinase NEK6

**DOI:** 10.1038/s41598-018-34471-y

**Published:** 2018-10-30

**Authors:** Marta De Donato, Benedetta Righino, Flavia Filippetti, Alessandra Battaglia, Marco Petrillo, Davide Pirolli, Giovanni Scambia, Maria Cristina De Rosa, Daniela Gallo

**Affiliations:** 1 Institute of Obstetrics and Gynecology, Università Cattolica del Sacro Cuore, Rome, Italy; 2 Department of Woman and Child Health, Fondazione Policlinico Universitario A. Gemelli, IRCCS, Rome, Italy; 3 Institute of Biochemistry and Clinical Biochemistry - Università Cattolica del Sacro Cuore, Rome, Italy; 4 Gynecologic and Obstetric Clinic, Department of Clinical and Experimental Medicine, University of Sassari, Sassari, Italy; 5 Institute of Chemistry of Molecular Recognition (ICRM) - CNR, Rome, Italy

## Abstract

The NIMA (never in mitosis, gene A)-related kinase-6 (NEK6), which is implicated in cell cycle control and plays significant roles in tumorigenesis, is an attractive target for the development of novel anti-cancer drugs. Here we describe the discovery of a potent ATP site-directed inhibitor of NEK6 identified by virtual screening, adopting both structure- and ligand-based techniques. Using a homology-built model of NEK6 as well as the pharmacophoric features of known NEK6 inhibitors we identified novel binding scaffolds. Twenty-five compounds from the top ranking hits were subjected to *in vitro* kinase assays. The best compound, i.e. compound **8** ((5Z)-2-hydroxy-4-methyl-6-oxo-5-[(5-phenylfuran-2-yl)methylidene]-5,6-dihydropyridine-3-carbonitrile), was able to inhibit NEK6 with low micromolar IC_50_ value, also displaying antiproliferative activity against a panel of human cancer cell lines. Our results suggest that the identified inhibitor can be used as lead candidate for the development of novel anti-cancer agents, thus opening the possibility of new therapeutic strategies.

## Introduction

Tumor cells accumulate alterations that result in uncontrolled proliferation and genomic instability, loss of normal cell-cycle control being actually a hallmark of human cancer^1^. Accordingly, numerous therapeutic strategies have been developed for targeting cell cycle in cancer, though the majority of available drugs also affect normal cells. The next generation of anti-mitotic therapies would thus ideally target cell-cycle features that are distinctive for tumor cells, as genomic instability and/or defective checkpoints in mitosis. Based on this assumption, the mitotic kinases governing centrosome dynamics and mitotic spindle function are potential targets for anticancer therapy^2^.

Eleven NIMA-related kinase (NEK) members have been identified in human genome (NEK1 to NEK11). Although their function still remains partially unknown, recent literature data support the hypothesis that some members of NEK family may play a role in mitotic progression; in detail, NEK2, NEK6, NEK7 and NEK9 have been reported to contribute to the establishment of the microtubule based mitotic spindle^3–5^.

Relevant to our research, protein level and activity of NEK6, identified in 2000 as a NIMA-related kinase highly similar to NEK7 (85% identity of the catalytic domain)^6^ have been shown to be increased in mitosis, whereas inhibition of its function has been reported to induce mitotic arrest, spindle defects, abnormal chromosome segregation and apoptosis^7,8^. Specifically, after phosphorylation by activated NEK9, NEK6 phosphorylates the kinesin Eg5 through which it regulates mitotic spindle formation^9^. NEK6 is also directly phosphorylated by CHK1 and CHK2, thus being identified as a novel direct target of the DNA damage checkpoint^10^. Notably, overexpression of NEK6 has been linked to many human diseases including liver, breast, lung, stomach, colon, larynx, ovary and prostate cancer^11–16^, and in line with these findings, we also recently showed that NEK6 is an independent unfavorable prognostic marker in ovarian cancer^11^. Taken together these data indicate that although the precise role in tumorigenesis remains unknown, NEK6 actually represents an attractive target for new anticancer therapies and inhibitors of NEK6 could be powerful compounds in the clinical setting. Several natural and synthetic molecules have been reported in literature with inhibitory activity on NEK6^12–16^. Recently, computer-aided drug design (CADD) strategies also have been attempted to rationally design novel NEK6 inhibitors, but *in silico*-identified hit compounds were not tested for activity in cell lines^17,18^. Overall, to date no inhibitor of NEKs has entered clinical trials for the treatment of cancer. The need to further investigate the role of NEK6 and the lack of adequate drug discovery efforts prompted us to identify novel compounds with NEK6 inhibitory activity. Here we report a search strategy for new putative inhibitors of NEK6 based on a sequential approach that involves a structure based virtual screening *via* docking simulations followed by the application of a pharmacophore-based screening to select the best candidates. Twenty-five compounds were identified and *in vitro* kinase assays demonstrated that the best compound (**8**) was able to inhibit NEK6 at low micromolar concentrations. Cellular assays subsequently demonstrated antiproliferative activity for the same compound.

## Results and Discussion

### NEK6 homology modeling

**Figure 1:**
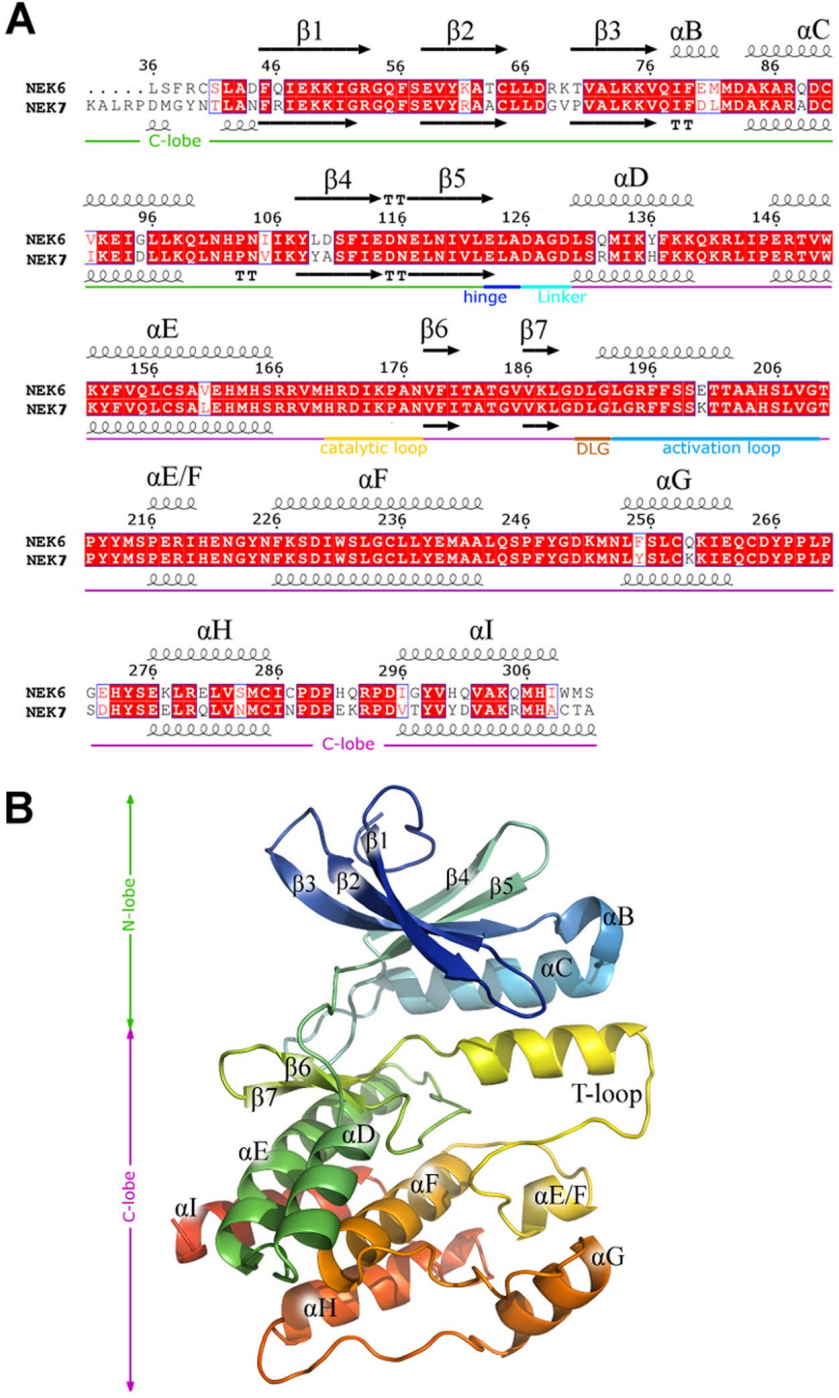
Homology model of NEK6. (**A**) Sequence alignment of NEK6 with NEK7 (2WQN). Secondary structure and functional elements are shown. Figure was prepared using ESPript web-based server (http://espript.ibcp.fr/ESPript/ESPript/)^61^. (**B**) Three-dimensional model structure of NEK6 in rainbow-colored solid ribbon representation.

NEK6 and the homologous NEK7 (313 and 302 residues, respectively) are the shortest members of the NIMA family, lacking the regulatory domain and consisting only of a catalytic domain with a very short N-terminal extension to the catalytic domain (NTE, residues 20–33) whose contribute to NEK7 activity was demonstrated^19^. Interestingly, although most of the functions described for NEK6 and NEK7 are very similar, the majority of NEK6 and NEK7 substrates identified to date are specific for one or other kinase^20^. To obtain a reliable structural model of NEK6, whose crystal structure has not yet been resolved, we applied two different homology modeling approaches: SWISS-MODEL and MODELLER. Initial screening for possible templates was performed using a PSI-BLAST^21^ analysis of the amino acid sequence of NEK6 against the PDB resolved structures. The currently available crystal structure of human NEK7, which shows the highest sequence identity (82%), was identified in both approaches as suitable template and the highest resolution structure was selected (PDB: 2WQN)^19^ (Fig. 1A). To select the models we relied on energy and stereochemical geometry. The overall stereochemical quality of the models was assessed by PROCHECK. The model structure obtained from SWISS-MODEL featured the majority of residues (98.0%) in the most favored region of the Ramachandran plot (85.9 and 12.1% in the core and allowed region, respectively), with only few residues localized in the generously allowed region (1.6%), and disallowed region of the diagram (0.4%). The corresponding percentages for the MODELLER structure were 98.4%, 1.2% and 0.4%. In order to verify whether the interaction energy of each residue with the remainder of the protein was negative (typically positive values correspond to tricky parts of a model) the protein structures were submitted to the ProSA web server available at https://prosa.services.came.sbg.ac.at/prosa.php. The low ProSA z-score values obtained (−7.37 and −7.33 for SWISS-MODEL and MODELLER models, respectively) are within the range of scores typically found for native proteins of similar size, thus confirming the good quality of our model. The reliability of the modeled fold was also checked with VERIFY-3D, which evaluates the compatibility of a given residue in a certain three-dimensional environment. A score below zero for a given residue means that the conformation adopted by that residue in the model is not compatible with its surrounding environment. The VERIFY-3D scores indicated that the 94.2% (SWISS-MODEL) and 86.6% (MODELLER) of the residues have a 3D-1D averaged score higher than 0.2 (Supplementary Information Figures S1 and S2). To measure the average distance between the backbone atoms of the generated models and template, the Cα-based superimposition RMSD values were calculated using Discovery Studio. Thus superposition of the generated models on the NEK7 template showed good overlap with RMSD values of 0.5 Å (SWISS-MODEL) and 0.6 Å (MODELLER), respectively. On the whole, no major differences were observed between the two generated model structures in these validation analyses. The protein, in inactive conformation^19^, revealed a bilobal fold consisting of a smaller N-terminal and a larger C-terminal lobe connected by a “hinge” (Leu124-Ala127). The N-lobe comprises a five-stranded β sheet and the “αC-helix” which shows an outward rotation characteristics of the inactive state, whereas the C-lobe is mostly α-helical (Fig. 1A,B). The C-terminal domain contains a flexible activation loop (T-loop), 20 amino acids (192–209) marked by a conserved Asp-Leu-Gly (“DLG”) motif at the start. Notably in the SWISS-MODEL structure, region G192-T202 adopts a α-helical conformation as observed in NIMA-related, EGRF and Src/Hck kinases^22–24^. This short helix, which has been already predicted for NEK6^25^, is not generated using MODELLER. For this reason the SWISS-MODEL-based homology-modeled structure of NEK6 was selected to be used in the subsequent docking simulations for virtual screening of inhibitors.

### Virtual screening

**Figure 2:**
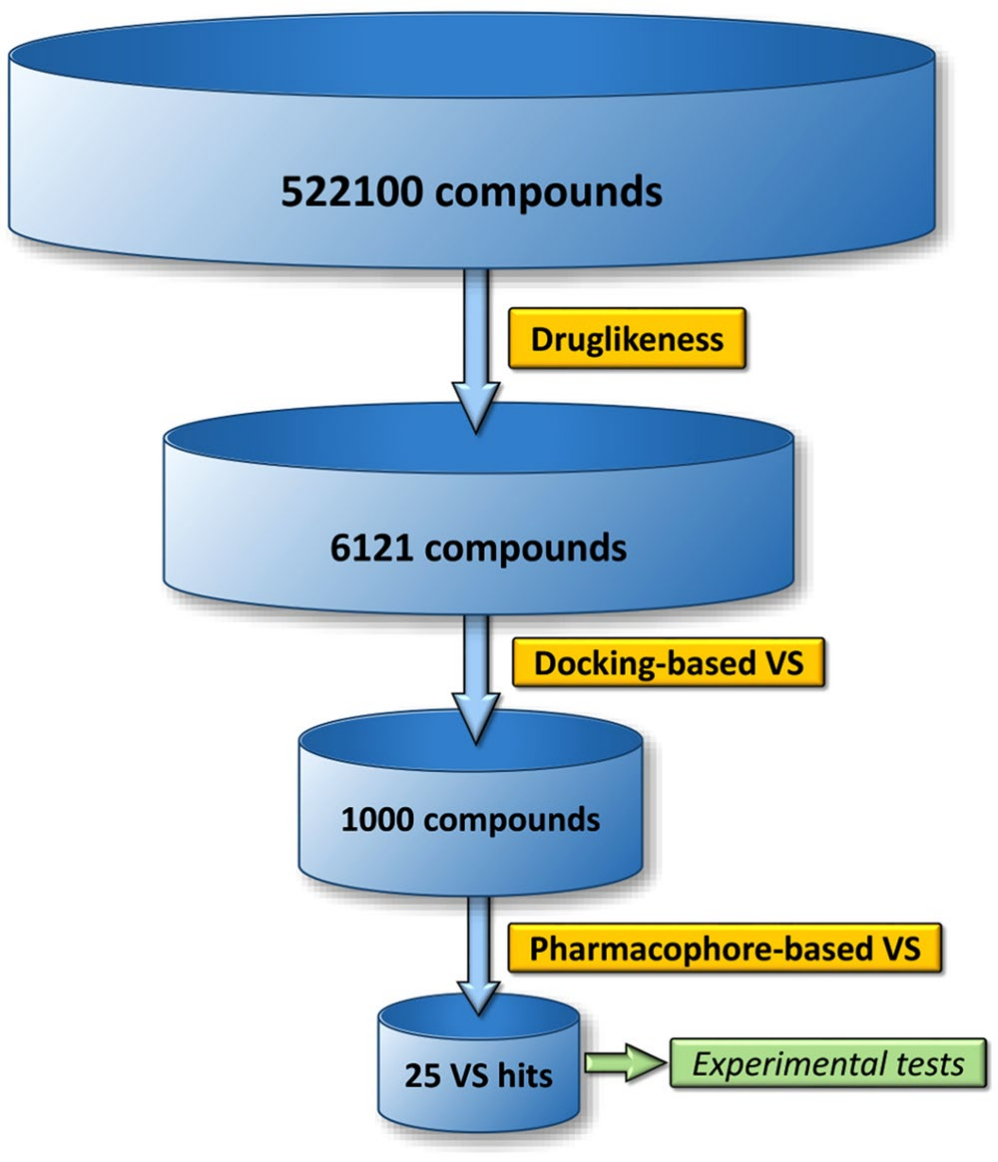
Flowchart of the virtual screening strategy.

**Figure 3:**
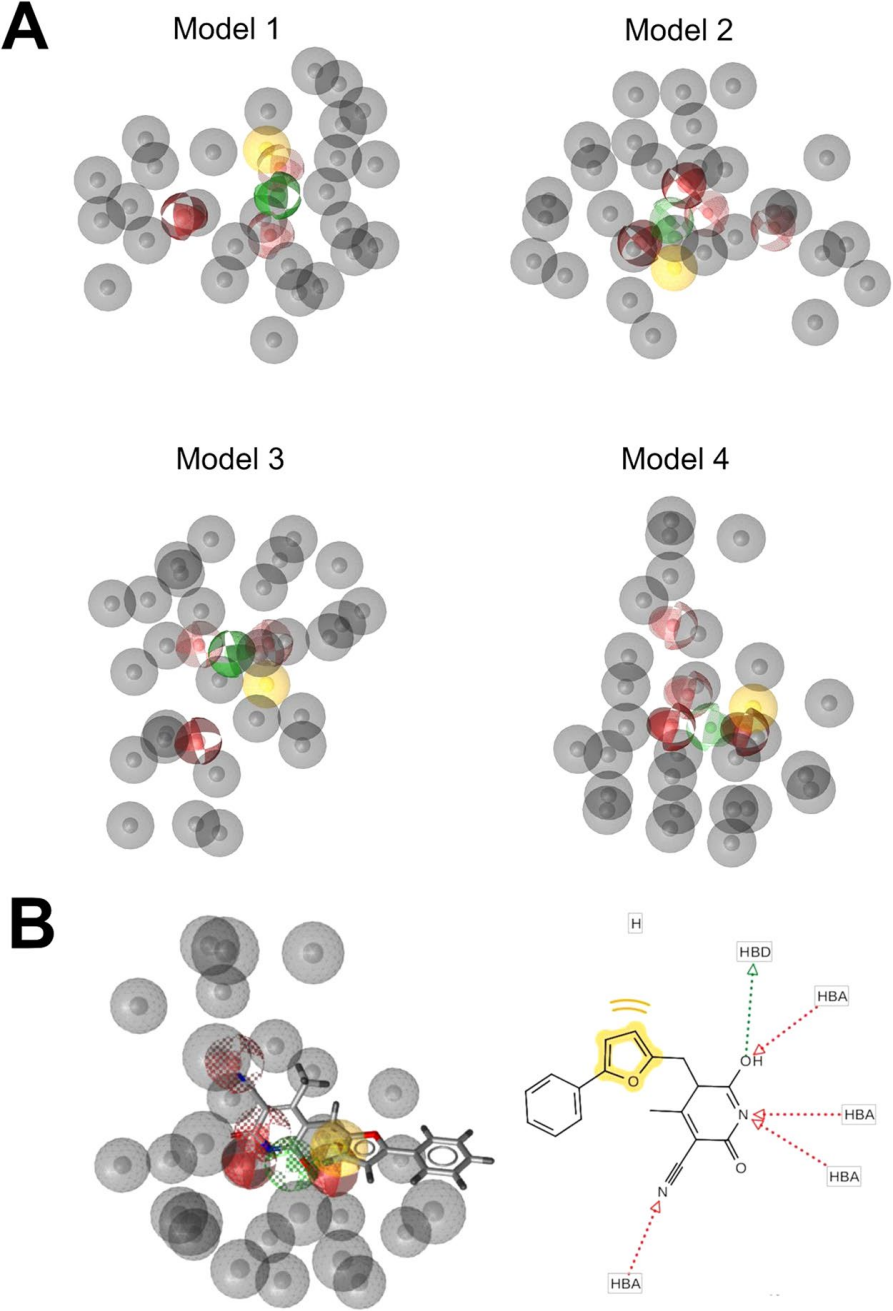
The top four pharmacophore models. (**A**) Model1-4, generated by LigandScout. The interactions were visualized in LigandScout with the following color code: HBA (red sphere), HBD (green sphere), H (yellow sphere) XVOLs (grey sphere). (**B**) Compound **8** superimposed to Model4 and in 2D representation.

To validate the reliability of the docking approach adopted in this work, AutoDock’s ability to predict binding poses and discriminate active from inactive compounds were explored. Validation of the accuracy of AutoDock was performed docking the crystallographic ADP cofactor, the unique available native co-crystallized ligand of NEK7, to the 2WQN binding site. A root mean square deviation (RMSD) of the of heavy atoms between the top-ranked pose and the crystallographic one of 1.5 Å was calculated, indicative of a successful scoring function^26^. A data set consisting of 11 NEK6 inhibitors from ChEMBL database (Supplementary Information Table S1) and 612 decoys generated with the Enhanced Directory of Useful Decoys resource (DUD-E)^27^ was used to evaluate sensitivity of the docking score using statistical parameters. The receiver operating characteristic curve (ROC), generated to reveal the overall screening performance, showed that the area under the curve (AUC) was 0.79 which represents a good predictive capacity (Supplementary Information Figure S3). We also measured the enrichment of inhibitors among the top-ranking scores of the simulated database through the enrichment factor (EF). We found an EF value of 3.2% for the first 20% of the database and 63.6% of active compounds in the top 20% of results. AutoDock 4.2 was then used for structure based virtual screening. At the time of this study Asinex and Maybridge libraries, as implemented in the ZINC12 database, consisted of 522100 compounds. To focus the virtual screening on compounds that could be promising for further development, we selected a subset of drug-like molecules using an online tool of ZINC12. This resulted in a subset of 6121 compounds. The general workflow of the multistep virtual screening approach implemented in this work is presented in Fig. 2. After selecting drug-like compounds, we employed a fast docking protocol to further filter the Asinex and Maybridges libraries using the generated homology model structure of NEK6. Based on the structural information provided by SiteMap and blind docking calculations (Supplementary Information Figures S4 and S5), structure based virtual screening targeting the ATP-binding pocket and the adiacent allosteric pocket^28^, was carried out. The rank of each compound was determined by the free energy of binding of the lowest energy conformation. The focused library consisting of the top 1000 AutoDock solutions was then screened against the four high ranking pharmacophore hypotheses generated with LigandScout 4.1 which is within the best performing algoritms in compound library enrichment^29^. No three dimensional structure has so far been reported for NEK6, therefore ligand-based pharmacophore models were developed for virtual screening. The eleven compounds with experimentally tested inhibitory activity to NEK6 available in ChEMBL database (Supplementary Information Table S1) were selected as a training set for pharmacophore model generation. Ten pharmacophore models (Model1–10) were generated by LigandScout, and the results are shown in Table 1. The features selected for this pharmacophore generation were hydrogen bond acceptor (HBA), hydrogen bond donor (HBD), hydrophobic (H) and exclusion volumes (XVOLs) into which a molecule is not allowed to protrude to avoid steric clashes. The default “pharmacophore fit and atom overlap” scoring function (values ranging from 0.79 to 0.74) was used to rank the generated 10 pharmacophore models. The hypotheses were validated using a data set of 12 known inhibitors and 650 decoys to determine how well they were capable to distinguish active from inactive compounds (Supplementary Information Table S2). The first four pharmacophore models, achieving the highest “pharmacophore fit and atom overlap” score (Table 1), were able to cover 91.7% of the active compounds and were selected for virtual screening of our library (Fig. 3A,B). For each pharmacophore model, the fifteen compounds with the highest fit values were selected. The hits were visually inspected for similarity to test a set of structurally diverse compounds. Finally, 25 virtual hits were selected, purchased and subjected to biological testing (Supplementary Information Table S3).

**Table 1:** Summary of the generated pharmacophore models.

Model	Pharmacophore Features				Pharmacophore fit and atom overlap score^a^
	H	HBA	HBD	XVOL	
1	1	3	1	27	0.7917
2	1	3	1	28	0.7911
3	1	4	1	25	0.7563
4	1	4	1	28	0.7556
5	1	4	1	30	0.7469
6	1	4	1	29	0.7458
7	1	4	1	27	0.7434
8	1	4	1	38	0.7430
9	1	4	1	30	0.7424
10	1	4	1	29	0.7403

Hydrogen bond donor (HBD); Hydrogen bond acceptor (HBA); Hydrophobic (H).

^a^Values normalized between 0 and 1.

### Identification of NEK6 inhibitors

**Figure 4:**
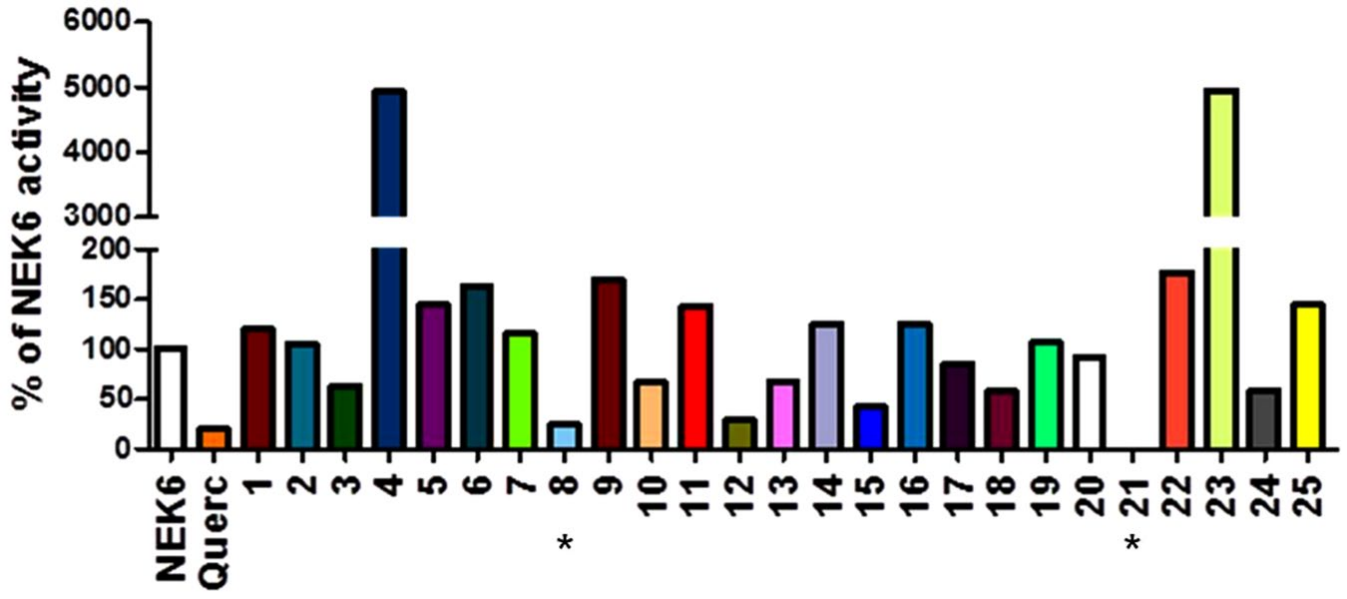
Screening of the selected twenty-five compounds at a single concentration by LANCE technology. Bar chart showing the percent of NEK6 activity, following treatment with the vehicle (NEK6 + vehicle) or the tested compounds at a concentration of 30 µM, using LANCE-Ultra NEK6 kinase assay. Quercetin was used as positive control of inhibition.

**Figure 5:**
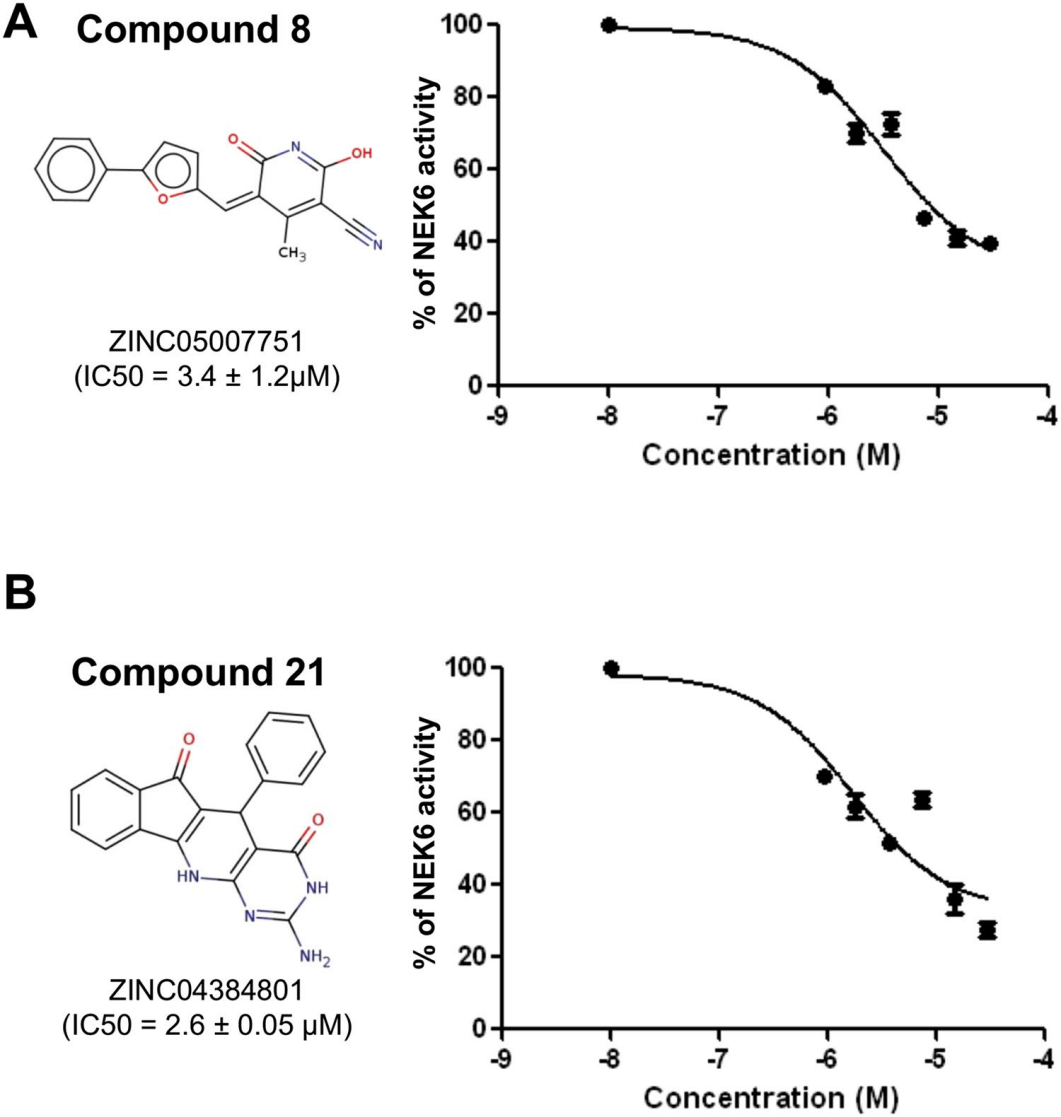
Identified inhibitors of NEK6 activity. Curves represent the percentage of NEK6 activity as a function of inhibitor concentration by LANCE-Ultra NEK6 kinase assay. (**A**) Compound **8** (ZINC05007751) inhibits NEK6 activity with IC_50_ value of 3.4 ± 1.2 µM; (**B**) compound **21** (ZINC04384801) inhibits NEK6 activity with IC_50_ value of 2.6 ± 0.05 µM. IC_50_ value derives by mean ± SEM of IC_50_ of 4 independent experiments. Each point of curve represents the mean of one triplicate ± SEM of one representative experiments.

**Figure 6:**
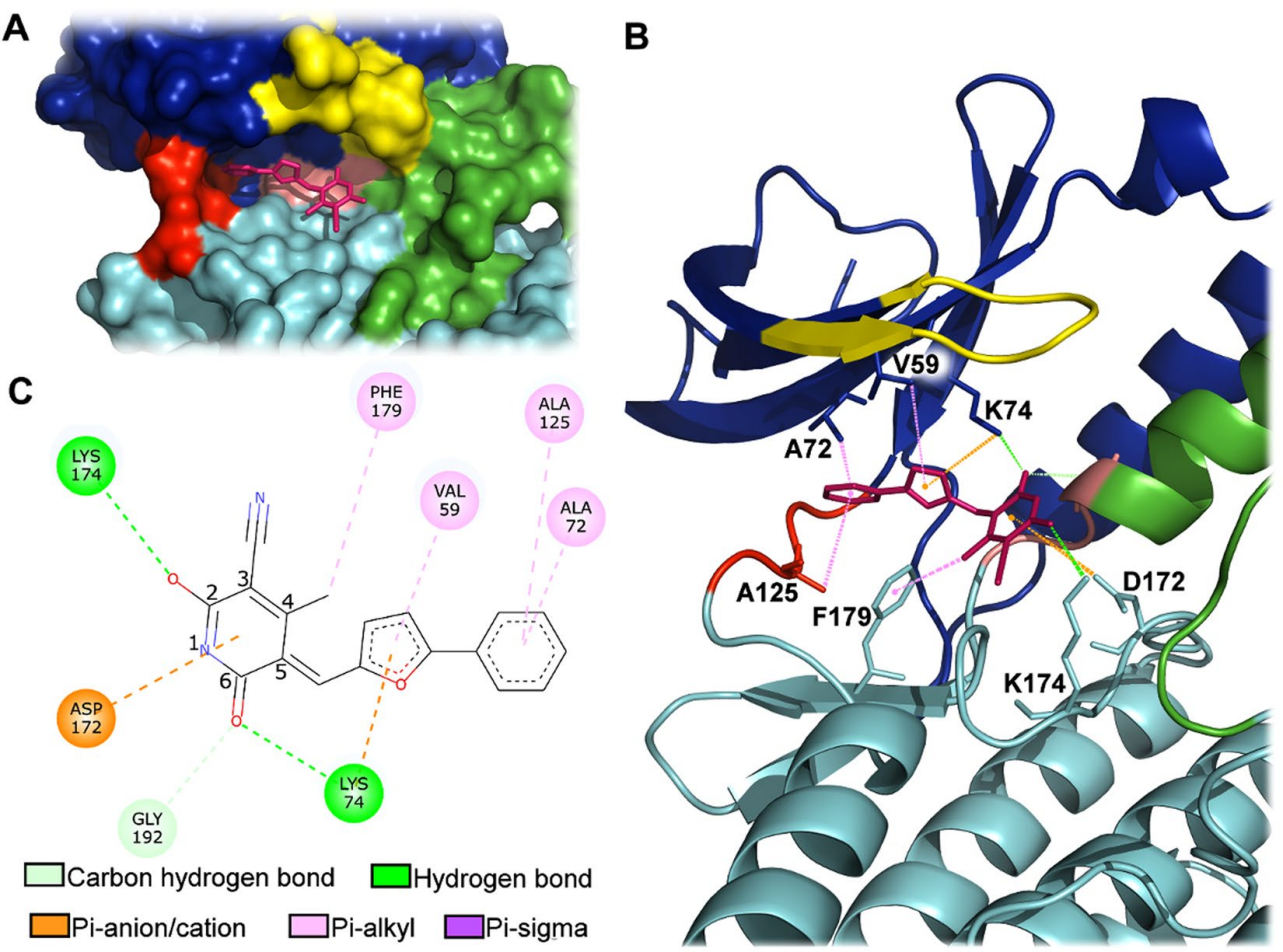
Predicted binding mode of compound **8**. (**A**) Solid surface of the ATP binding pocket of NEK6. (**B**) Three-dimensional representation. (**C**) Two-dimensional interaction plot. The N-lobe is coloured in blue, the C-lobe in light-blue, the activation loop in green, the DLG motif in pink, the Gly-rich loop in yellow and the hinge-region in red.

The twenty-five compounds resulting from virtual screening were firstly tested for the ability to inhibit the enzymatic activity of NEK6 at a single concentration of 30 µM, using LANCE technology. Quercetin, able to inhibit a broad panel of kinase, was used as positive control of inhibition^12,30^. This initial screening allowed the identification of two compounds determining >70% NEK6 inhibition at 30 µM, i.e. compound **8** (6-hydroxy-4-methyl-2-oxo-5-[(5-phenyl (2-furyl)) methylene] pyridine-3-carbonitrile), and **21** (6-amino-2-phenyl-5,7,9-triazatetracyclo[8.7.0.0^3,^^8^ 0^11,^^16^]heptadeca-1(10),3(8),6,11,13,15 hexaene-4,17-dione) (Fig. 4). Using the same technology, we determined IC_50_ values of 3.4 ± 1.2 and 2.6 ± 0.05 µM (IC_50_ ± SEM) for **8** and **21**, respectively (Fig. 5A,B). A second technique (Off-chip Mobility shift assay) was then used to confirm these data, results showing IC_50_ values of 13.8 µM for compound **8** and 49.8 µM for compound **21** (CARNA Biosciences Study ID: CBS170097, Supporting Information, Figure S6). Thus, while substantially confirming IC_50_ value for **8**, this latter technical approach gave a value significantly higher for compound **21**. Indeed, as it is widely accepted^31,32^, factors related to differences in methodologies of detection and experimental conditions (e.g. buffer composition, pH, enzyme quality, incubation temperatures, ATP concentrations) might account for the differences observed in IC_50_ values obtained for **8** (i.e. 3.4 vs 13.8 µM). On the other hand, discrepant values measured for **21** (i.e. 2.6 vs 49.8 µM) were suggestive of a low intrinsic activity and/or solubility of the molecule: due to this reason, we focused on **8** as a potential lead compound for further investigation. The possible binding mode of compound **8** in the ATP-binding site of NEK6 as well as nonbonded interactions across the complex interface are shown in Fig. 6. Compound **8** may use its phenyl ring and furyl ring to form hydrophobic Pi-alkyl interactions with Ala72 (strand β3), Ala125 (hinge region) and Val59 (strand β2) of the ATP binding site (Fig. 6B). Notably, the dihydropyridinic ring of compound **8**, besides interacting with Lys174 of the catalytic loop, forms a short C^α^–H···O hydrogen bond with Gly192 of the conserved DLG motif (Fig. 6B). This is particulary relevant when considering that C–H···O hydrogen bonds play an important role in the ligand-binding process^33–35^.

### Kinase selectivity

The structure of NEK6 exhibits a significant identity to NEK7 and thus developing NEK6-selective inhibitors is arduous because of the highly similar (85% identical) catalytic domains^3^. Therefore, we decided to evaluate the selectivity of compound **8** against a panel of NEK kinases at 10 µM, using the technique of Off-chip Mobility shift assay. This concentration was chosen according to the guidelines proposed by Knight and Shokat, since the probability of off-target effects increases dramatically above 10 µM^36^. Furthermore, ATP concentration was set to approximate the K_m,ATP_ for each kinase, so that the resulting selectivity profile directly reflects the intrinsic affinities of the inhibitor^37^. Notably, results obtained showed that compound **8** is very selective against NEK1 and NEK6, with comparable inhibition of 47% and 42.5%, respectively while no significant activity was observed against NEK2, NEK7, and NEK9 (≤0, ≤0 and 3.1% respectively). Considering the high similarity between NEK6 and NEK7, these results may be related to the presence of a glutamic acid rather than lysine in position 200 of NEK6 activation loop (Fig. 1). A major consequence of this substitution may be the redistribution of surface charges at the flexible activation loop resulting in an altered electrostatic potential and ability of NEK6 kinase to interact with other molecules.

### Antiproliferative activity of compound 8

**Figure 7:**
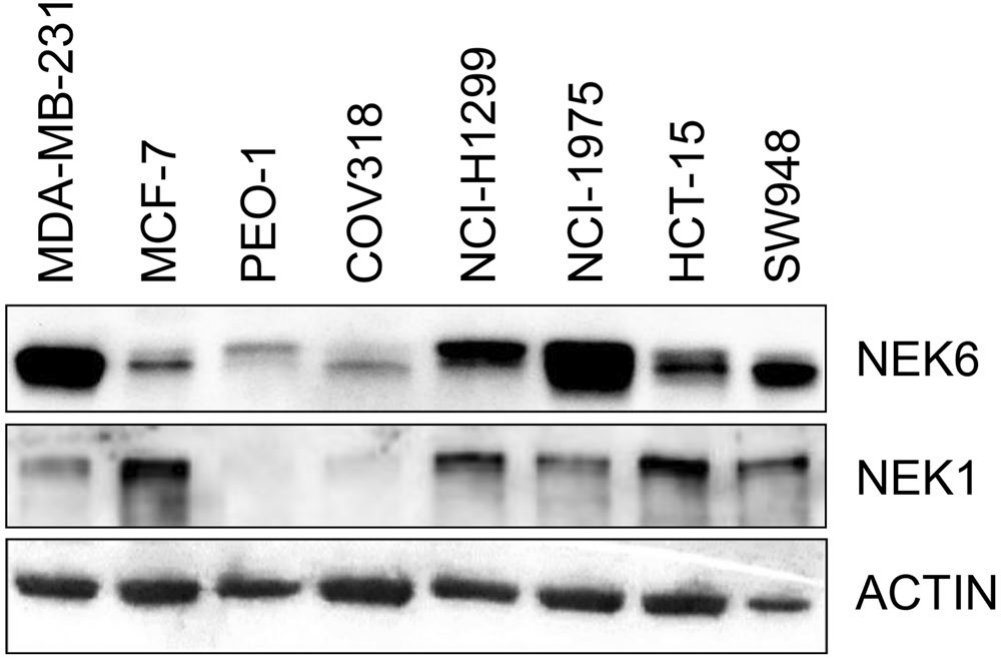
Western blot analysis for NEK6 and NEK1 on a panel of human cancer cell lines. Western blot showing NEK6 and NEK1 expression levels on a panel of cancer cell lines representative of four solid tumors: MDA-MB231 and MCF7 (breast cancer), PEO1 and COV318 (ovarian cancer), NCI-H1299 and NCI-H1975 (lung cancer), HCT-15 and SW948 (colon cancer). Actin immunoreaction was used as loading control. The cropped blots are used in the figureand full-length blots are presented in Supplementary Figure S7.

A panel of cancer cell lines representative of human tumors, including breast (MDA-MB-231, MCF-7), ovary (PEO1, COV318), lung (NCI-H1975, NCI-H1299) and colon (HCT-15, SW948), were firstly analyzed for NEK6 and NEK1 expression. Western blot analysis showed that both NEK1 and NEK6 protein were present in the selected experimental models, although at a different extent (Fig. 7). Therafter, the ability of compound **8** in inhibiting cell growth was tested in this panel of tumor cell lines. Results obtained demonstrated that **8** was able to inhibit the growth of MDA-MB-231, PEO1, NCI-H1299 and HCT-15 with IC_50_ value below 100 µM (Table 2). Activity did not correlate with the expression level of the target proteins (i.e. NEK6 and NEK1), this suggesting that other factors may contribute in driving cytotoxicity. Indeed, it is possible that the different susceptibilities of human cancer cell lines to compound **8** might also reflect the genomic diversity of human cancer (Supplementary Information Table S4), in keeping with the notion that cell lines faithfully recapitulate oncogenic alterations identified in tumors and that many of these associate with drug sensitivity/resistance^38^.

**Table 2:** *In vitro* evaluation of the cytotoxic effect of compound **8** on a panel of cancer cell lines.

Cancer cell lines	IC_50_ Values (µM ± SEM)
	Compound 8
**MDA-MB-231** (breast)	65 ± 15
**MCF-7** (breast)	NA
**HCT15** (colon)	98.5 ± 0.5
**SW948** (colon)	NA
**PEO1** (ovary)	44 ± 6.5
**COV318** (ovary)	NA
**NCI-H1975** (lung)	NA
**NCI-H1299** (lung)	87.8 ± 10

IC_50_: 50%-inhibitory concentration or compound concentration required to inhibit tumor cell proliferation by 50%; SEM: standard error of the mean of at least two independent experiments; NA = not active.

### Compound 8 induces perturbation of PEO1 cell cycle

**Figure 8:**
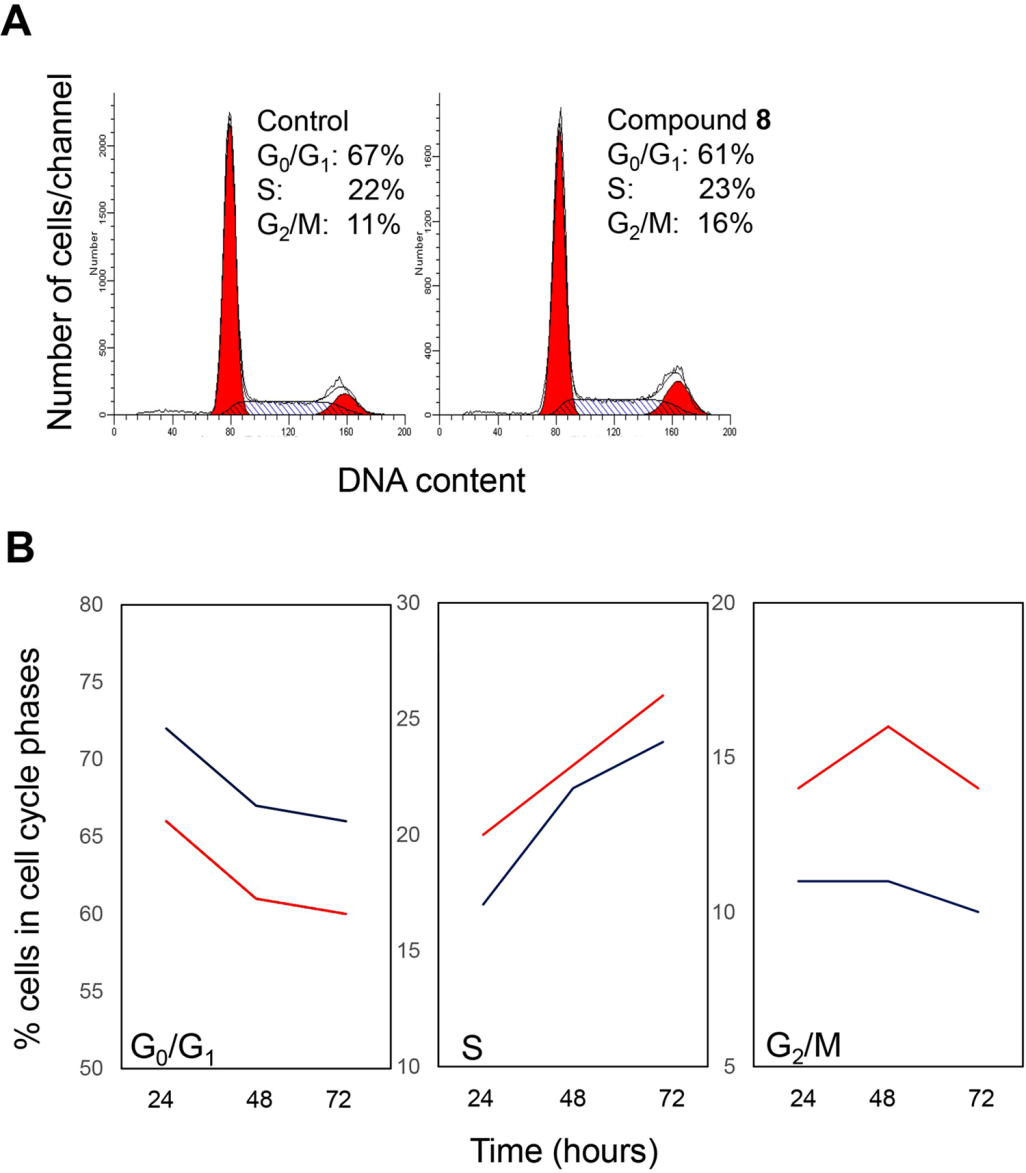
Cell cycle analysis by flow cytometry. (**A**) representative monoparametric DNA content histograms obtained from PEO1 cells harvested at 48 hours (when differences in comparison to control became more evident). Discrimination of G0/G1, S and G2/M subcompartments of the cell cycle were computed by the MofdFit LT software. Left panel: control PEO1 cells; right panel: PEO1 cells treated with compound **8**. (**B**) Time course analysis of percentage of PEO1 cells in the different phases of cell cycle assessed at 24, 48 and 72 hours from treatment. Results are from one representative experiment out of two independent experiments performed with similar results. Blue line: control cells; red line: compound **8**-treated cells.

In order to assess whether the ability of compound **8** to inhibit cell proliferation was accounted for by cell cycle-related changes in cell division, we analysed the progression of PEO1 cells through the cell cycle by flow cytometry at 24, 48 and 72 hours from treatment. PEO1 was selected since, in our experimental conditions, was the cell line showing the highest sensitivity to **8**. Cell cycle analysis showed that compound **8** induced PEO1 cell cycle perturbations, with a prominent accumulation of cells in the G_2_/M phases of the cell cycle, in the absence of measurable effect on the S phase, and with a consequent reduction of cells in the G_0_/G_1_ compartment of cell cycle. The effect was already measurable at the first experimental time point (24 h) and remained stable over time (Fig. 8). Thus, these data provide evidence that compound **8**-induced decrease in PEO1 cell proliferation is associated with perturbation of cell passage through the G_2_/M phases of cell cycle. These data are in keeping with previous results on requirement of a functional NEK6 for cells entering mitosis and on arrest of cells in the M phase of cell cycle upon NEK6 silencing^8^. Notably, more in general, it is widely accepted the key role of NEK kinases activity in cell cycle progression^3^.

### Combination study of compound 8 with anticancer drugs on ovarian cancer cells PEO1

Platinum- and taxane-based regimens represent the standard of care for adjuvant treatment in epithelial ovarian cancer^39^. Therefore, the possibility to use the tested compound in combination with standard cytotoxic drug was investigated in PEO1, cell line representative of high grade serous ovarian cancer harbouring a germline mutation in BRCA2, a gene encoding a protein essential for DNA repair by homologous recombination (HR)^40,41^ (Supporting Information, Table S4). Cisplatin IC_50_ value obtained in PEO1 in our experimental conditions was 7.9 ± 0.65 µM (mean ± SEM), in line with literature data^26,42^. Compound **8** showed synergism with cisplatin, resulting in a significant reduction of cisplatin IC_50_ from 7.9 ± 0.65 to 0.1 ± 0.01 µM, with combination **8** (44 µM) + cisplatin (10 µM) exhibiting the greatest synergistic effect (Table 3). From a mechanistic point of view, our results are coherent with the evidence suggesting that, besides NEK1, also NEK6 may have a role in DNA damage response, with the identification of several novel NEK6 interactors involved in this process^43^. Given this background, the synergistic effect on PEO1 between **8** and cisplatin could derive by genomic instability resulting from different processes, including (a) PEO1 inability to repair DNA damage by homologous repair and nucleotide excision repair^40,44^, (b) inhibition of the DNA damage repair activity exerted by NEK1 and, possibly, by NEK6 and, finally (c) by the impairment of NEK6 and mitotic functions that may lead to further abnormal centrosome functions, spindle defects and chromosome mis-segregations.

**Table 3:** *In vitro* evaluation of the cytotoxic effect of compound **8**/cisplatin combination on PEO1 cells.

Cell line	Drug		C.I. (mean ± SEM)	Effect of combination
	Cisplatin (µM)	Compound 8 (µM)		
PEO1	0.1	44	0.81 ± 0.08	Synergism
	1	44	1.08 ± 0.26	Nearly additive
	2	44	1.01 ± 0.12	Nearly additive
	5	44	1.03 ± 0.18	Nearly additive
	10	44	0.69 ± 0.01	Synergism

C.I.: Combination Index. C.I. < 0.1 Very strong synergism; 0.1–0.3 Strong synergism; 0.3–0.7 Synergism; 0.7–0.85 Moderate synergism; 0.85–0.90 Slight synergism; 0.90–1.10 Nearly additive; 1.10–1.20 Slight antagonism; 1.20–1.45 Moderate antagonism; 1.45–3.3 Antagonism; 3.3–10 Strong antagonism; >10 Very strong antagonism; SEM: standard error of the mean of three independent experiments.

With regard to taxane, the suggested role of NEK kinases in microtubule dynamics regulation supported our investigation to evaluate the effect of a combination between compound **8** and paclitaxel on PEO1 cells. Indeed, previous findings have shown that NEK6 and NEK7 may directly regulate microtubule organization^45^, an aspect that makes them attractive targets for drugs that would be complementary to microtubule targeting agents^2^. The IC_50_ value for paclitaxel on PEO1 cells was within the range reported in literature^42,46^. Results obtained showed a slight synergistic effect at 1 nM paclitaxel, shifting IC_50_ paclitaxel from 7.0 ± 0.001 to 0.64 ± 0.057 nM (Table 4). However, at higher paclitaxel concentrations antagonistic effects were observed. Although this is not an unusual finding, since synergism and antagonism can be different at different dose levels^47^, it certainly requirer further investigation to properly identify the best combination drug schedule. Results from the combination studies are in line with our previous findings demonstrating that stable NEK6 silencing in ovarian cancer cells produced a modest but significant increase of cisplatin and paclitaxel sensitivity, while a stable overexpression slightly increased drug resistance^11^.

**Table 4:** *In vitro* evaluation of the cytotoxic effect of compound **8**/paclitaxel combination on PEO1 ovarian cancer cell line.

Cell line	Drug		C.I. (mean ± SEM)	Effect of combination
	Paclitaxel (nM)	Compound 8 (µM)		
PEO1	1	44	0.87 ± 0.023	Slight synergism
	10	44	1.05 ± 0.003	Nearly additive
	100	44	4.68 ± 0.132	Strong antagonysm
	1000	44	29.58 ± 0.353	Very strong antagonism
	10000	44	287.79 ± 0.001	Very strong antagonism

C.I.: Combination Index. C.I. < 0.1 Very strong synergism; 0.1–0.3 Strong synergism; 0.3–0.7 Synergism; 0.7–0.85 Moderate synergism; 0.85–0.90 Slight synergism; 0.90–1.10 Nearly additive; 1.10–1.20 Slight antagonism; 1.20–1.45 Moderate antagonism; 1.45–3.3 Antagonism; 3.3–10 Strong antagonism; >10 Very strong antagonism; SEM: standard error of the mean of three independent experiments.

## Conclusions

Here, we employed *in silico* screening techniques to search for novel NEK6 inhibitors. The best identified hit, i.e. ((5Z)-2-hydroxy-4-methyl-6-oxo-5-[(5-phenylfuran-2-yl)methylidene]-5,6-dihydropyridine-3-carbonitrile) is able to inhibit NEK6 at micromolar order of magnitude. Interestingly, **8** shows selective inhibition for NEK6 and NEK1, while not acting on NEK7; the identification of a compound able to inhibit NEK6 with respect to NEK7 is an important achievement, considering that NEK6 and NEK7 have the highest degree of sequence identity and differ only for one residue in the active site^48^. Compound **8** shows antiproliferative activity against a panel of human cancer cell lines, and displays a synergistic effect with cisplatin and paclitaxel in a BRCA2 mutated ovarian cancer cell line, this supporting a possible use for personalized therapy. On the whole, the newly discovered inhibitor deserves consideration for further development by SAR studies to optimize its activity.

## Methods

### Molecular modeling

We implemented standard computational strategies to construct the tridimensional model of human NEK6, since its experimental structure is not yet available. The sequence was obtained from the UniProt database (UniProt accession number Q9HC98) and the modeling was performed using two different homology modeling approaches: the fully automated structure modeling method implemented in the SWISS-MODEL workspace (http://swissmodel.expasy.org//SWISS-MODEL.html)^49^ and the program MODELLER^50^, as implemented in DiscoveryStudio 4.5 (Dassault Systèmes BIOVIA), which generates a protein model through the satisfaction of spatial restraints. The crystallographic structure of human NEK7 (PDB code 2WQN)^19^ was identified by PSI-BLAST^21^ as tertiary structural template showing 82% sequence identity. The generated models were evaluated using the programs PROCHECK, VERIFY3D and ProSA-Web^51–53^.

### Structure based virtual screening

The first virtual screening step was carried out using docking simulations to generate a library containing molecules having appropriate structural complementarity with the search zone defined around the ATP binding site in NEK6. The atomic coordinates of NEK6 obtained from the homology modeling were used as the receptor model in the virtual screening with docking simulations using AutoDock 4.2.6^54^. To verify the predictive ability of AutoDock scoring function for this target, a data set consisting of 11 NEK6 inhibitors from ChEMBL database (http://www.ebi.ac.uk/chembl/) (Supplementary Information Table S1) and 612 decoys generated with the Enhanced Directory of Useful Decoys resource (DUD-E)^27^ was used. Enrichment calculation and receiver operator characteristic (ROC) curve analysis were performed using the Enrichment Calculator tool of Schrödinger suite (Schrödinger LLC). Compounds from suppliers Asinex and Maybridge were downloaded from the ZINC12 database^55^ selecting the drug-like subset^56^. The selected descriptors were the molecular weight (150 < MW < 500), the hydrophobicity (−4 < xlogP < 5), the net charge (−5 < NC < 5), the number of rotable bonds (RB < 8), the number of H-bond donors (HBD < 10), the number of H-bond donors acceptors (HBA < 10), the polar surface area (Å^2^) (PSA < 150), the polar desolvation (kcal/mol) (−400 < PD < 1), the apolar desolvation (kcal/mol) (−100 < AD < 40). The filtering procedure yielded 6121 compounds. Both the NEK6 receptor and the small molecules were prepared for docking using the AutoDockTools (ADT) software package^57^. Polar hydrogen atoms were added and Kollman charges and atomic solvation parameters were assigned to the protein. For all ligands, Gasteiger charges were assigned, non-polar hydrogen atoms merged and all torsions were allowed to rotate during docking. The auxiliary program AutoGrid generated the grid maps. In the absence of co-crystallized ligands, prediction of binding sites in NEK6 was carried out using SiteMap^58^ (Schrödinger) and AutoDock blind docking of the 11 ChEMBL inhibitors. The SiteMap primary site (highest site score of 0.861), was utilised as reference for generating the focused AutoDock grids. The grids, one for each atom type in the ligands (A, C, HD, N, NA, OA, SA, Cl, I, F, S, Br) plus one for electrostatic interactions, were computed covering the putative ATP binding site using 60 × 60 × 60 grid points with a spacing of 0.375 Å. A Lamarckian genetic algorithm was chosen, and default parameters were used except “Number of GA runs”, “Population size”, and “Maximum number of evaluations”, which were set to 10, 50, and 2.5 × 10^5^. Results were clustered according to all-atom RMSD (root mean square deviation) with a tolerance of 2 Å and representative binding mode was defined as the lowest-energy complex of the cluster with the largest population.

### Pharmacophore model generation and ligand based virtual screening

LigandScout 4.1^59^, *via* a ligand-based strategy, was used for 3D pharmacophore generation, refinement and virtual screening. This approach searches for a common feature pattern that is shared in an active ligand set and considers the conformational flexibility of the ligands. The training set consisted of the eleven ChEMBL compounds with inhibitory activities to NEK6. Twelve kinase inhibitors from the DrugKiNET database (www.drugkinet.ca) were taken as a test set for pharmacophore model validation using a decoy set of 650 inactive compounds assembled from DUD-E database^27^ (Supplementary Information Table S2). Excluded volumes representing the sterically occupied regions by the receptor, were taken into account to increase the selectivity of the model. All LigandScout parameters were kept default and the models were ranked based on the “pharmacophore fit and atom overlap scoring function”. The four best-fitting models exhibiting good score values were selected and applied to a 3D virtual focused library of 1000 compounds represented by the top 1000 AutoDock solutions.

### NEK6 kinase assay

Compounds (i.e. **1** to **25**) (Molport, Riga, LV-1011, Latvia) were dissolved in DMSO 100% as 10 mM stock and stored at −20  C. Activity of the compounds as NEK6 inhibitors was tested using LANCE NEK6 Ultra Kinase Assays protocol (U-TRF #25 technical note, PerkinElmer, Monza, MB). Compounds were first tested at 30 µM and incubated with 4 nM NEK6 (Carna, Chuo-ku, Kobe, Japan, # 05-130), 50 nM ULight-p70 S6K Peptide (PerkinElmer # TRF0126) and 100 μM ATP (Sigma-Aldrich, Saint Louis, U.S.A, # A2383) at room temperature. Kinase reactions were terminated after 90 minutes by the addition of EDTA and the signal was read with the Enspire plate reader (PerkinElmer) in TR-FRET mode (excitation at 320 nm and emission at 665 nm) after 60 minutes. Inhibition of NEK6 activity was calculated as percentage with respect to the control sample (100% of activity). Quercetin was used as positive control (Molport). For IC_50_ determination, the selected compounds **8** (Molport-002-933-483) and **21** (Molport-000-911-820) were tested at dilutions ranging from 30 µM to 0.9375 μM. For each compound the dose inhibiting 50% of NEK6 activity (IC_50_) was calculated using the GraphPad Prism 5.0 Software (San Diego, CA, USA).

### Off-chip Mobility Shift Assay (MSA)

The inhibitory activity of compounds **8** and **21** on NEK6 was also verified with MSA technique (Off-chip Mobility shift assay) (CARNA Biosciences Study ID: CBS170097, CARNA Biosciences, Kobe). For IC_50_ determination, compounds were tested at dilutions ranging from 100 to 0.003 μM, and incubated with NEK6 enzyme, 1000 nM CDK7 peptide, 5 mM Mg and Km app/Bin: 69/75 μM ATP. PKR Inhibitor was used as positive control. For profiling study against NEK kinases (i.e. NEK1, NEK2, NEK6, NEK7, NEK9) (CARNA Biosciences Study ID: CBS170098), **8** was tested at 10 μM and ATP concentration approximating Km for each kinase. Assay condition for kinases are available on “Kinase profiling book” downloading by www.carnabio.com (https://www.carnabio.com/output/pdf/ProfilingProfilingBook_en.pdf).

### Cell cultures

The following human carcinoma cell lines were used for the study: MDA-MB-231 and MCF-7 (breast cancer; ATCC, Sesto San Giovanni, MI), PEO1 and COV318 (ovarian cancer; ECACC, Salisbury, UK and ATCC), HCT-15 and SW948 (colon cancer; Public Health England, Salisbury, UK and ATCC), NCI-H1975 and NCI-H1299, (lung cancer; ATCC). Cells were routinely tested free of mycoplasma (MycoAlert mycoplasma detection kit, LONZA, Rockland, ME, USA) and validated by STR (Short Tandem Repeat) DNA profiling (BMR Genomics srl, PD). Cells were grown following supplier indication, in a fully humidified atmosphere of 5% CO_2_/95% air. All the reagents were purchased from Sigma-Aldrich (St. Louis, MO), if not otherwise specified.

### Western blot analysis

Western blot analysis was carried out as previously described, following cell incubation in lysis buffer containing 20 mM Tris-HCl pH 7.4, 5 mM EDTA, 150 mM sodium chloride, 1% glycerol e 1% Triton X-100, in the presence of a cocktail of both protease and phosphatase inhibitors^11^. The following antibodies were utilized: anti-NEK6 antibody (1:5000; #ab109177 Abcam, Cambridge, UK), anti-β-actin antibody (1:5000, #A5441 Sigma-Aldrich, St. Louis, MO), anti-NEK1 antibody (1:200, #sc-398813 Santa Cruz Biotechnology, Heidelberg, Germany).

### Cell-based cytotoxicity assays

Cells were seeded (40.000 cells/ml for HCT-15 and MCF-7, 100.000 cells/ml for the remaining lines) in a 96 well black plate (PerkinElmer). Compound **8** was added in quadruplicate, 24 h after plating, at concentrations ranging from 6 × 10^−6^ M to 1.9 × 10^−4^ M; cisplatin and paclitaxel (Sigma-Aldrich St. Louis, MO) were used from 1 × 10^−9^ M to 1 × 10^−5^ M. Growth inhibition was evaluated after 72 h by using ATPlite kit (PerkinElmer). To assess efficacy of drug combinations, PEO1 cells were treated with a fixed dose of **8** (IC_50_) and increasing cisplatin or paclitaxel concentrations. The percentage of growth inhibition with respect to control and the dose inhibiting 50% of cell growth (IC_50_) were calculated for each cell line by statistical analysis software, Graphpad 5.0 (San Diego, CA, USA). Compounds showing an IC_50_ greater than 100 μM were considered not active. Combination indices (CI) were calculated using Compusyn program for multiple drug effect analysis based on the equation of Chou-Talalay^47,60^.

### Cell cycle analysis by flow cytometry

PEO1 cell cycle perturbations induced by compound **8** (used at its IC_50_ concentration) were evaluated by standard flow cytometry methods. Briefly, at the end of each incubation period adherent cells were trypsinized, harvested and washed with cold phosphate-buffered saline several times. Cells were then counted, gently fixed in 70% v/v cold ethanol, adding the ethanol dropwise to the cell pellet while vortexing, and incubated at −20 °C for no longer than 7 days. Prior to DNA staining, fixed cells were spun down and treated with RNase (100 µg/ml) for 10 mins to ensure that only DNA was stained. 1 × 10^6^/ml cells were then stained with propidium iodide (PI, 0.5 mg/ml) and stored at +4 °C overnight. The day after, stained cells were subjected to flow cytometry. Flow cytometry analysis was performed using the 6-parameter flow cytometer (2 scatter and 4 fluorescence signals) EPICS-XL (Beckman Coulter). A minimum of 30,000 cells of interest were acquired for each sample at a low flow rate (<200 events/sec). Analysis of cell cycle perturbation was performed by the ModFit LT software (Verity software house). Pulse shape processing was used to exclude cell doublets from the analysis.

## Electronic supplementary material

Supplementary Information
