# Supplementary material for: Identification and antitumor activity of a novel inhibitor of the NIMA-related kinase NEK6
Source: Sci Rep. 2018 Oct 30;8:16047. doi: 10.1038/s41598-018-34471-y (PMC6207720; doi:10.1038/s41598-018-34471-y)
Supplement: Supplementary file 1 — Supplementary Information [file 41598_2018_34471_MOESM1_ESM.pdf]

Supplementary Information

Identification and antitumor activity of a novel inhibitor of the NIMA-related kinase NEK6

Marta De Donato[1,2], Benedetta Righino[3], Flavia Filippetti[1,2], Alessandra Battaglia[1], Marco Petrillo[1,4], Davide Pirolli[5], Giovanni Scambia[1,2], Maria Cristina De Rosa[5,*], Daniela Gallo[1,2]

[1]Institute of Obstetrics and Gynecology, Università Cattolica del Sacro Cuore, Rome, Italy;

[2]Department of Woman and Child Health, Fondazione Policlinico Universitario A. Gemelli, IRCCS, Rome, Italy;

[3]Institute of Biochemistry and Clinical Biochemistry - Università Cattolica del Sacro Cuore, Rome, Italy.

[4]Gynecologic and Obstetric Clinic, Department of Clinical and Experimental Medicine, University of Sassari, Sassari, Italy;

[5]Institute of Chemistry of Molecular Recognition (ICRM) - CNR, Rome, Italy

*Corresponding author:
Maria Cristina De Rosa
Institute of Chemistry of Molecular Recognition - CNR,
c/o Università Cattolica del Sacro Cuore, L.go F. Vito 1, 00168 Rome, Italy
Email: mariacristina.derosa@icrm.cnr.it
phone: +39 06 30155135

Figure S1. Structural evaluation of the SWISS-MODEL model by using (A) Verify-3D, (B) PROCHECK, (C, D) ProSA-Web.

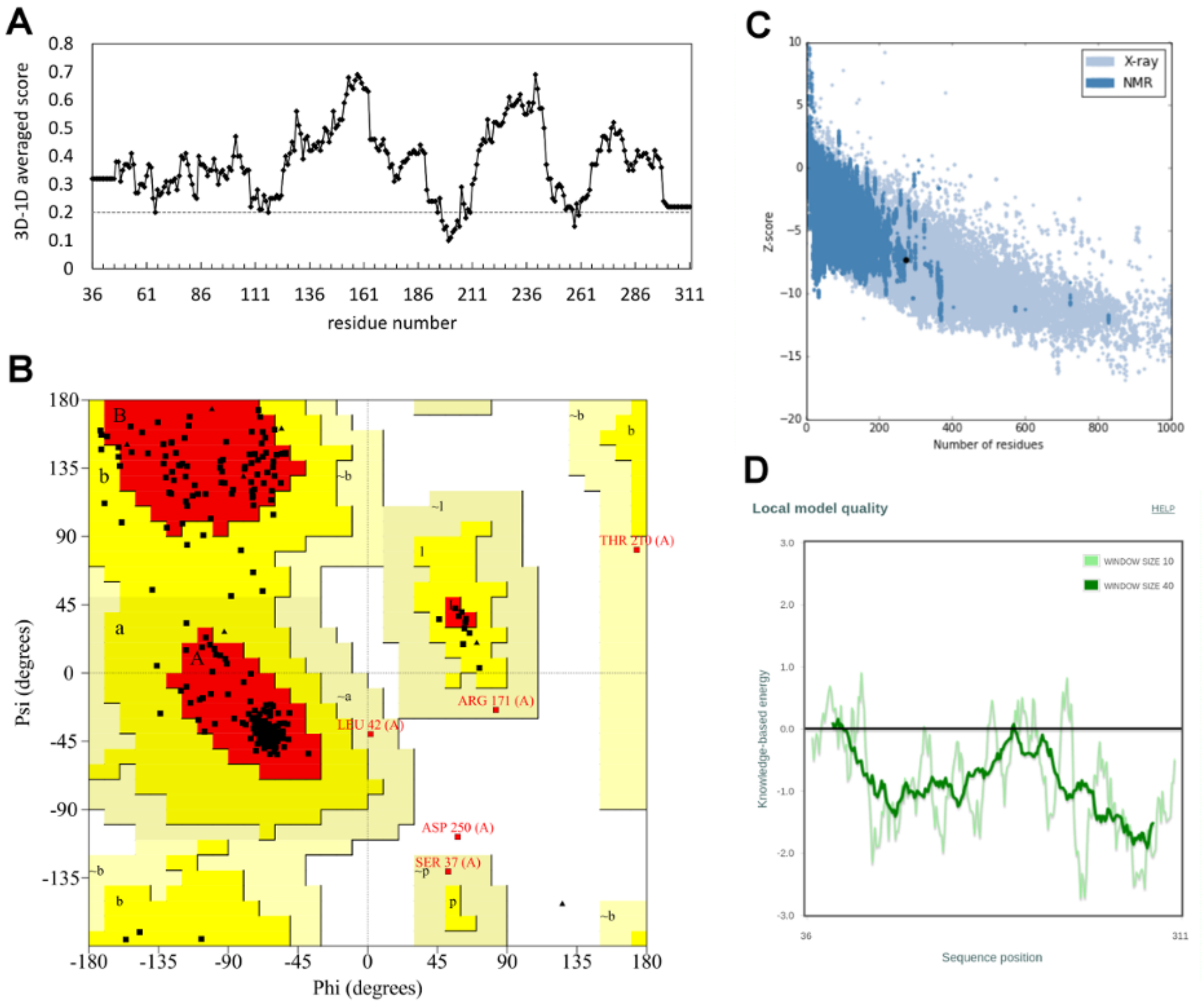

Figure S2. Structural evaluation of the MODELLER model by using (A) Verify-3D, (B) PROCHECK, (C, D) ProSA-Web.

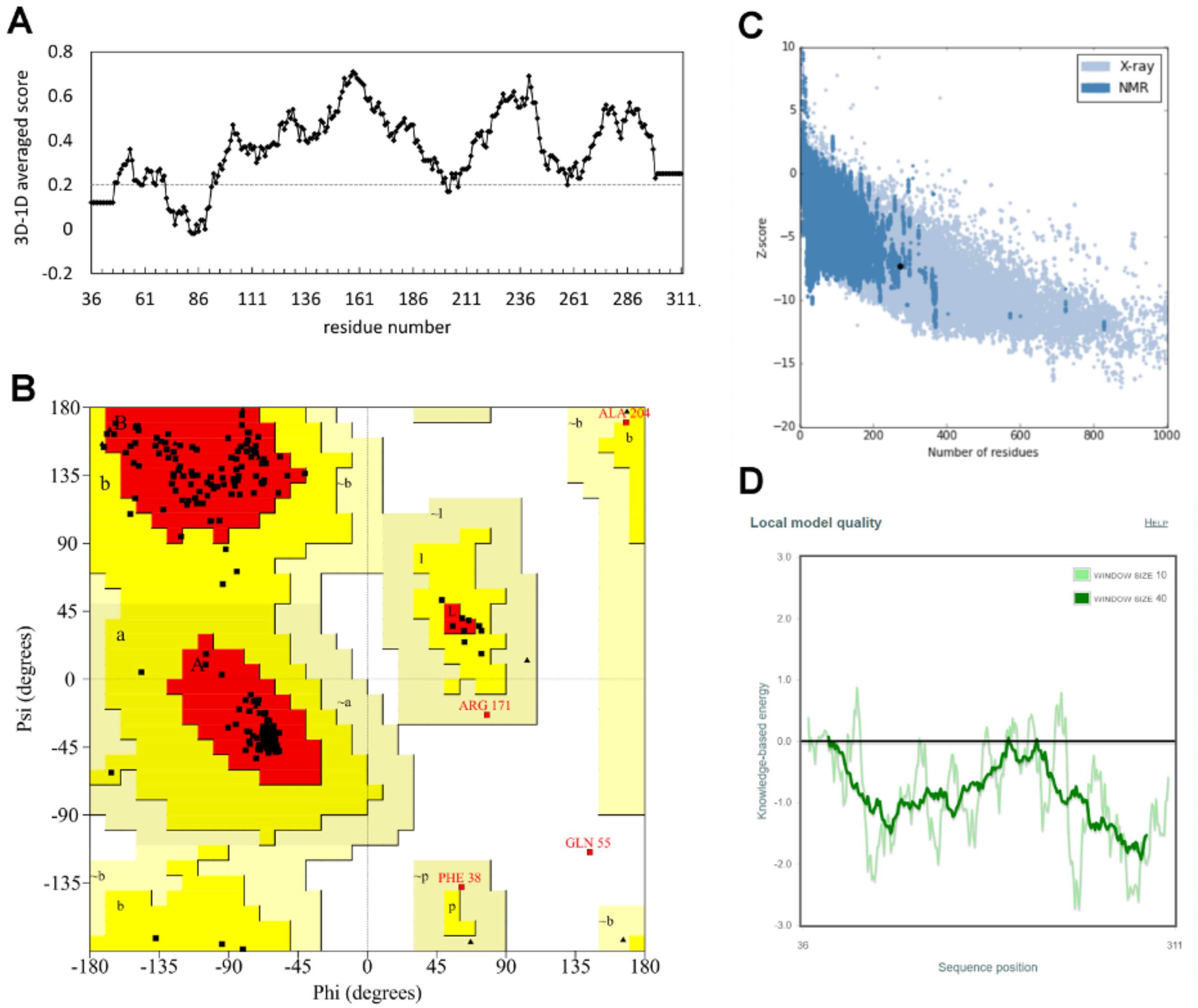

Figure S3. Receiver Operating Characteristics (ROC) curve obtained from the screening of 11 molecules with known activities and 612 decoys.

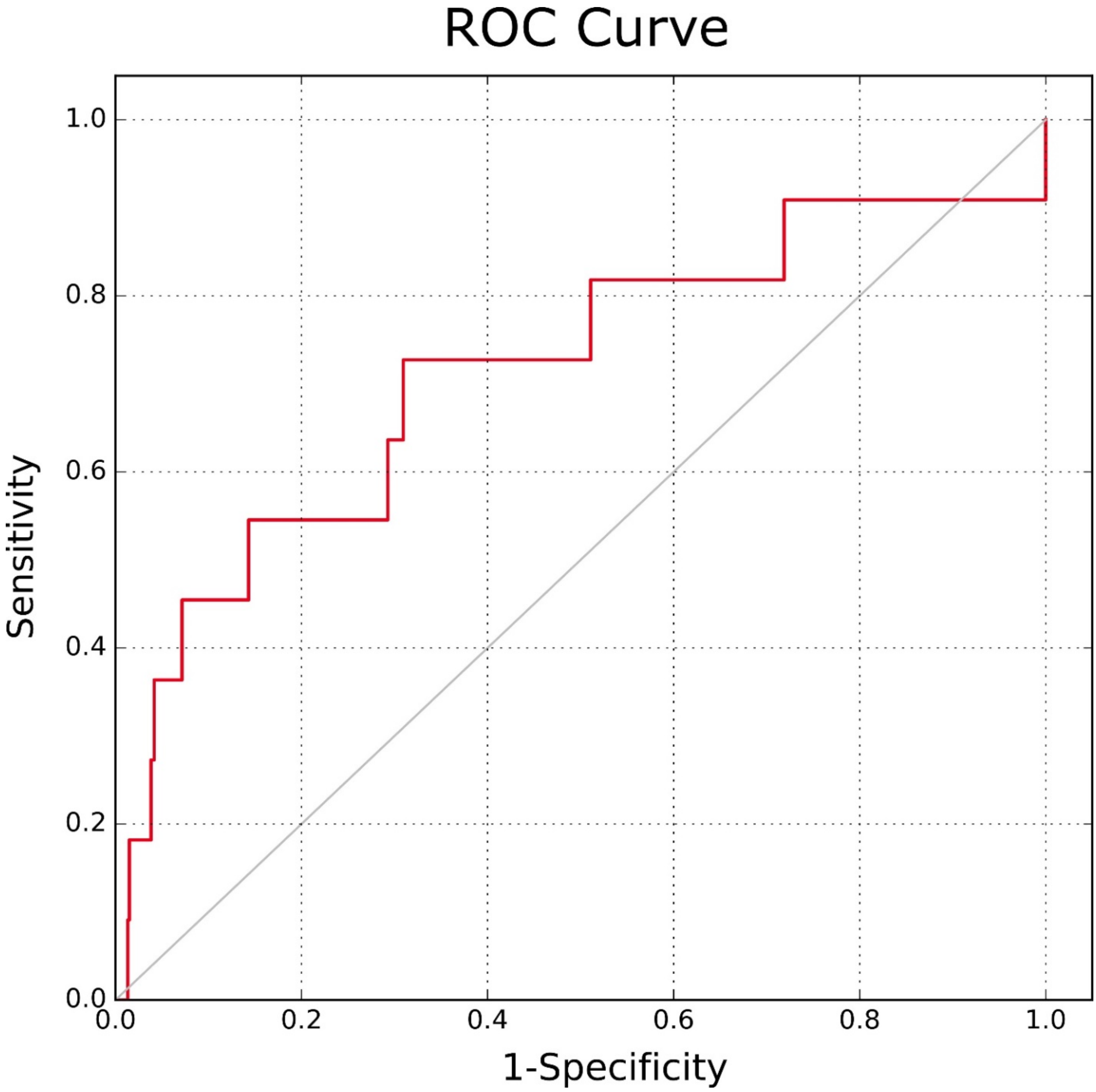

Figure S4. Binding site identification by SiteMap and blind docking experiments. The location of the primary binding site identified by SiteMap is highlighted with blue spheres (A). It is a large binding site where the 11 ChEMBL compounds preferentially bind NEK6 as demonstrated by blind docking experiments. The lowest energy conformation of the most populated cluster for each ChEMBL is shown (B). Solid solvent accessible surface representation of NEK6 is displayed: the N- and C-terminal domains (light and dark grey, respectively), the Gly-rich loop (green), the DLG motif (red), the hinge (orange) and the activation loop (violet).

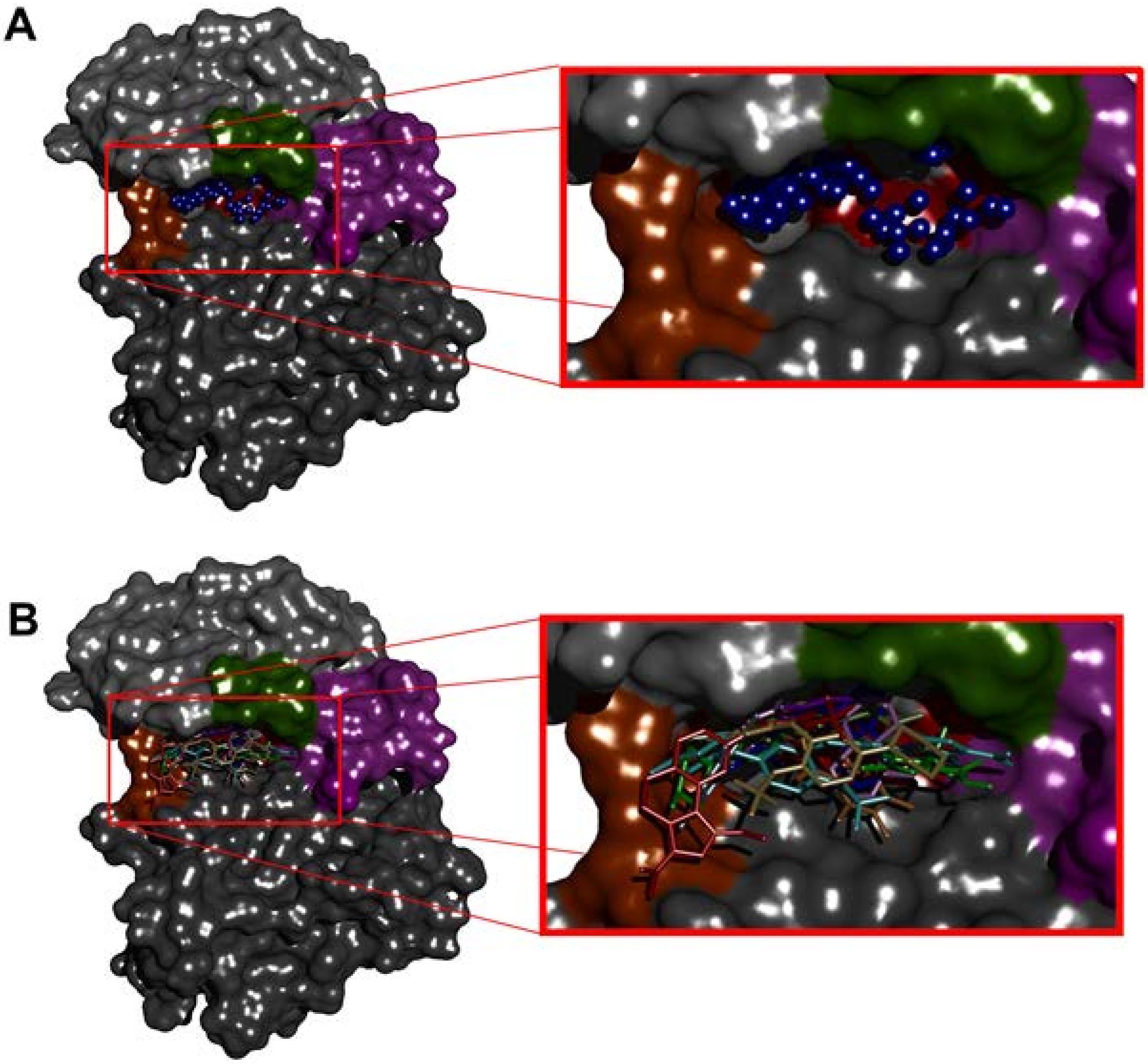

Figure S5. Histograms of clusters for blind docking experiments. The size of the clusters versus the lowest binding free energy of the poses in the cluster is shown. For all the 11 ChEMBL compounds the most populated cluster (red vertical bar) is positioned in the ATP-binding pocket. A clustering cutoff of 10Å was used.

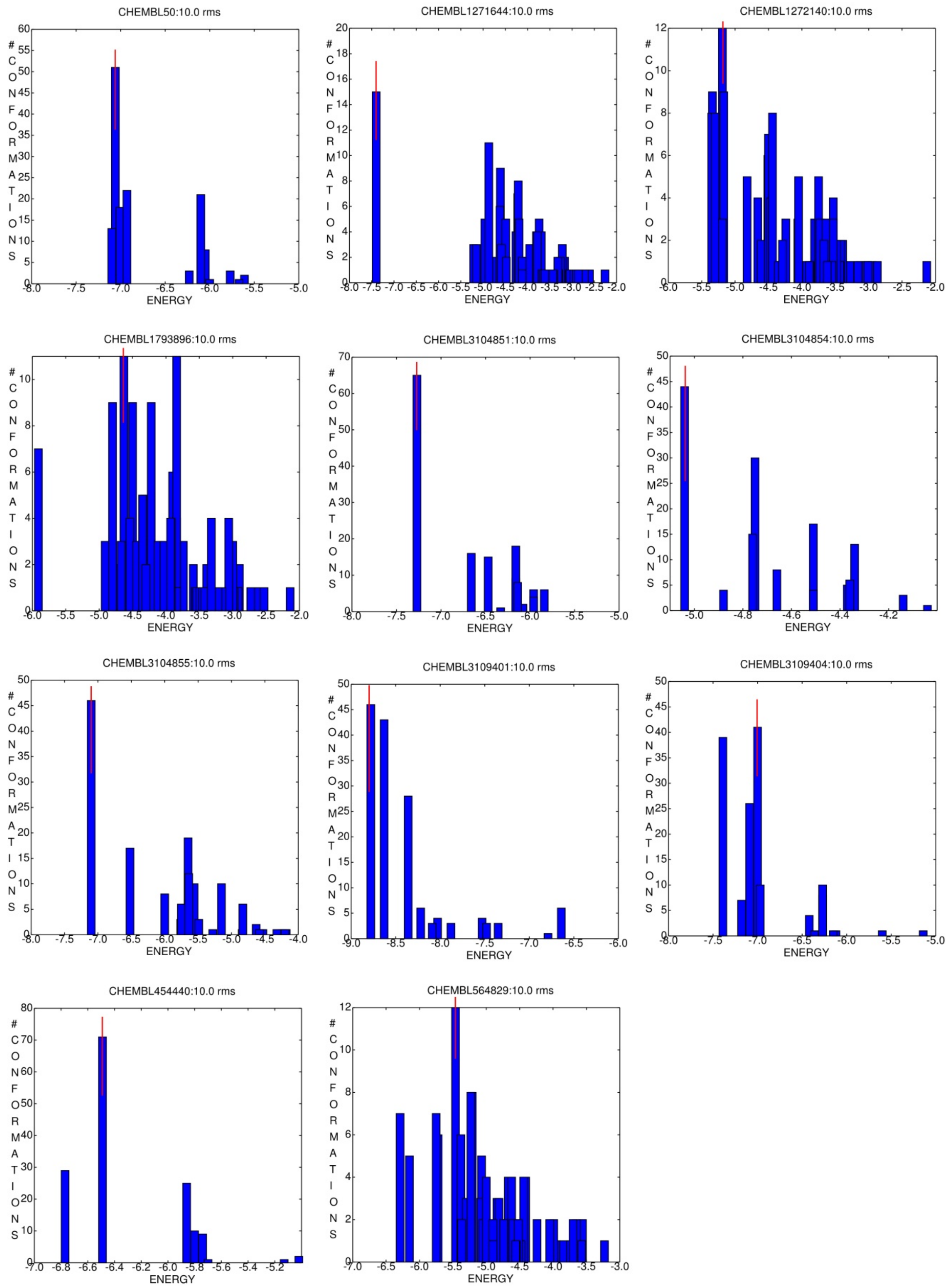

Figure S6. IC_{50} calculation by CARNA study CBS-170097 with Off-chip Mobility shift assay technology. Curves represent the percentage of NEK6 inhibition as a function of compound **8 (A)** and compound **21 (B)** concentration.

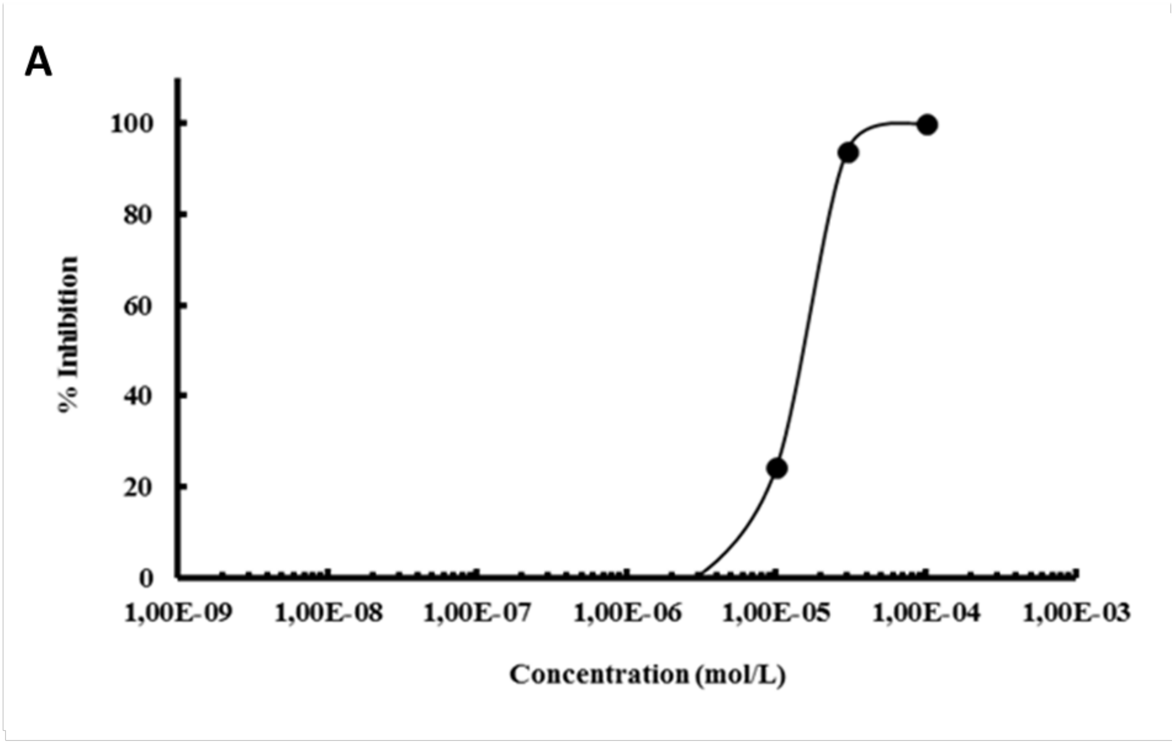

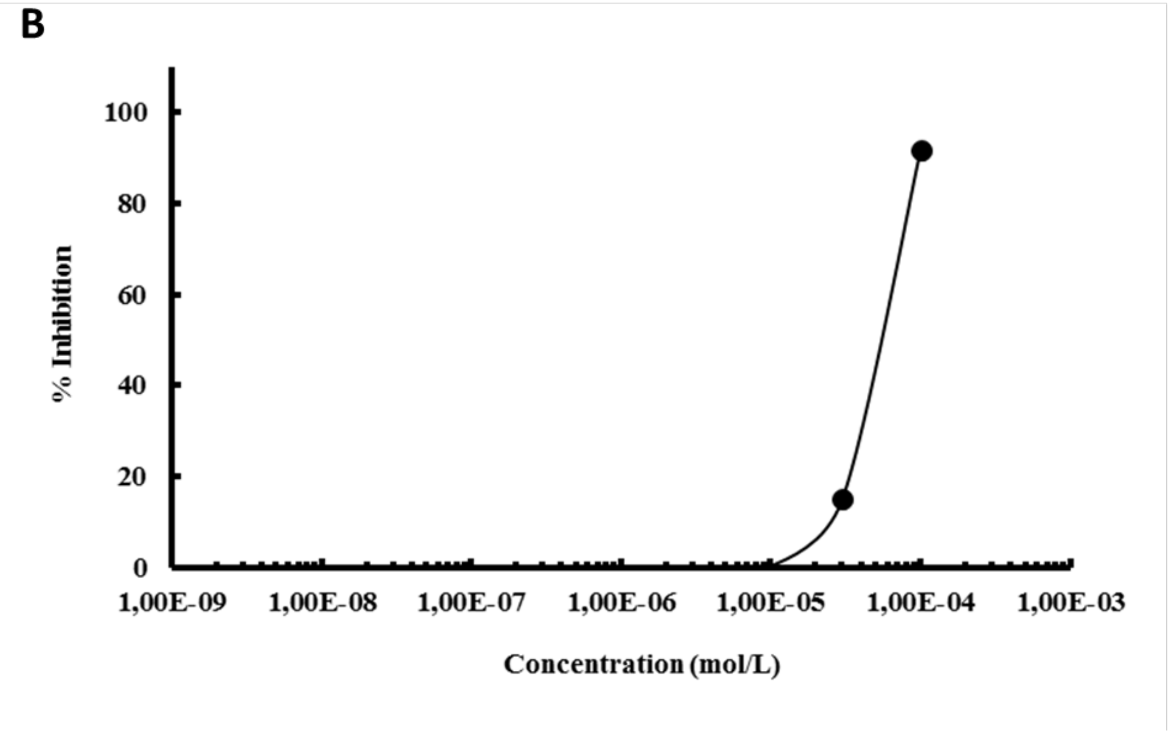

Figure S7. Full length blots of Figure 7. **(a-c)** NEK6**:** multiple exposures with the different exposure times; **(d)** actin acquisition; **(e-f)** NEK1: multiple exposures with the different exposure times. Brightness was adjusted during processing these blots.

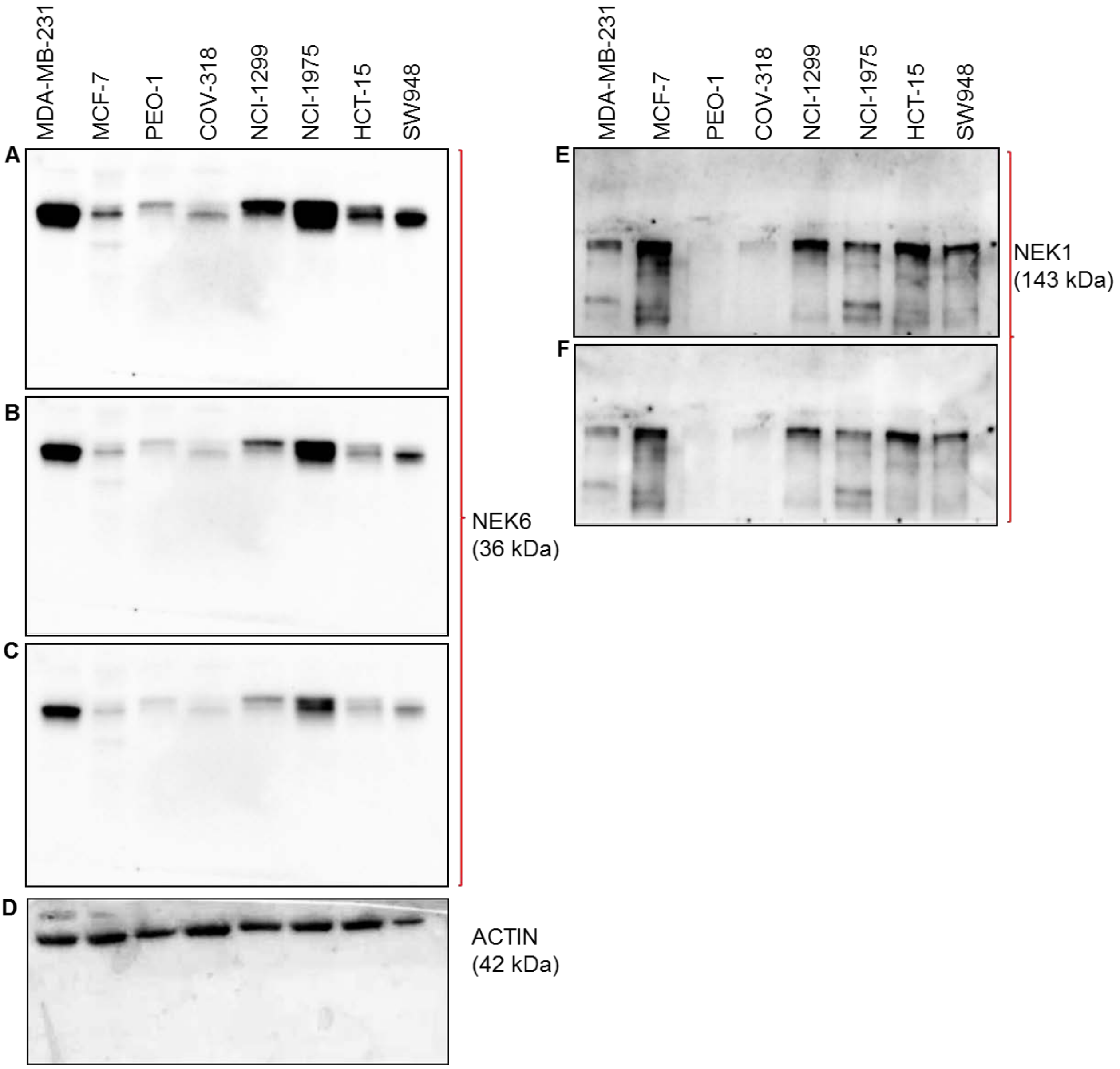

Table S1. Structures of training-set compounds and their experimental IC_{50} values

1<br>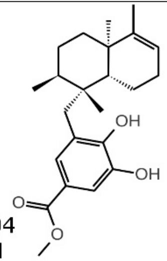<br>CHEMBL3109404<br>IC50 = 7.72 uM	2<br>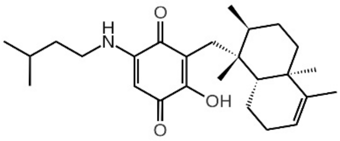<br>CHEMBL3109401<br>IC50 = 90.8 uM	9<br>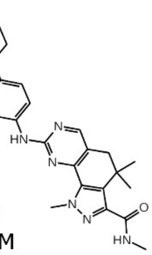<br>CHEMBL564829<br>IC50 = 1.065 uM
3<br>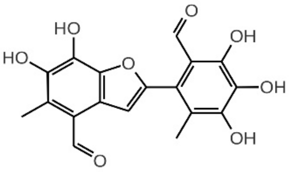<br>CHEMBL3104855<br>IC50 = 1.85 uM	4<br>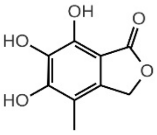<br>CHEMBL3104854<br>IC50 = 13.6 uM	10<br>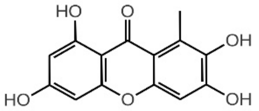<br>CHEMBL454440<br>IC50 = 67.8 uM
5<br>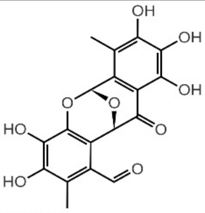<br>CHEMBL3104851<br>IC50 = 0.52 uM	6<br>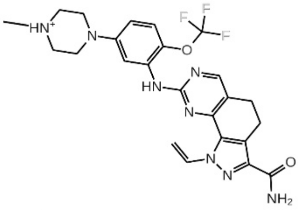<br>CHEMBL1793896<br>IC50 = 0.336 uM	11<br>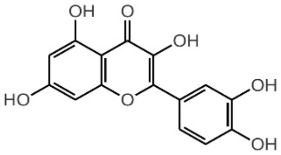<br>CHEMBL50<br>IC50 = 4.23 uM
7<br>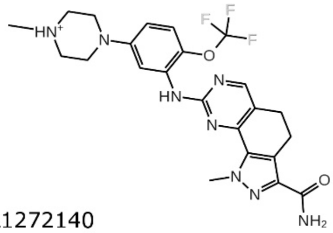<br>CHEMBL1272140<br>IC50 = 1.664 uM	8<br>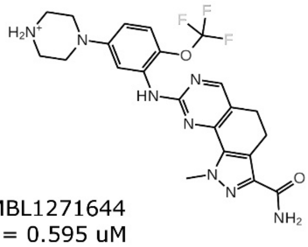<br>CHEMBL1271644<br>IC50 = 0.595 uM	

Table S2. Structures of testing-set compounds and their experimental IC_{50} values. The acronym "CID" stands for PubChem compound identifier.

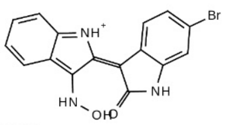<br>CID: 5287844<br>IC50 (nM) < 4	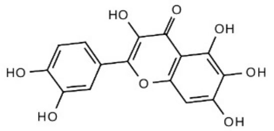<br>CID: 5281680<br>IC50 (uM) < 6	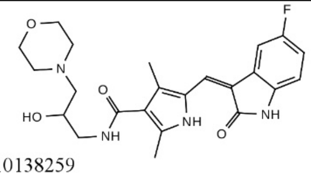<br>CID: 10138259<br>Kd (uM) = 0.6
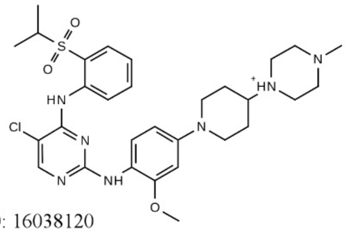<br>CID: 16038120<br>Kd (uM) =1.3	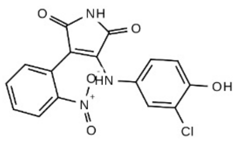<br>CID: 4210951<br>IC50 (uM) < 6	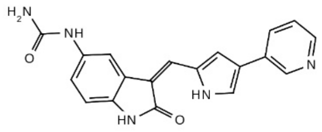<br>CID: 11588244<br>IC50 (uM) < 8
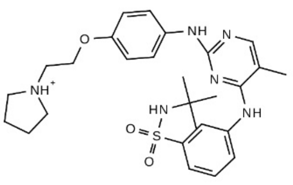<br>CID: 16722836<br>IC50 (uM) =0.12	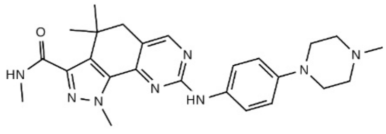<br>CID: 16718576<br>IC50 (uM) =1.065	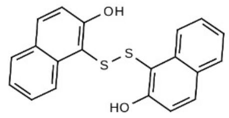<br>CID: 521106<br>IC50 (uM) < 0.8
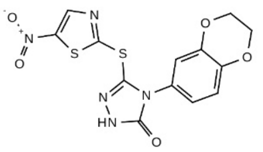<br>CID: 2747117<br>IC50 (uM) < 6	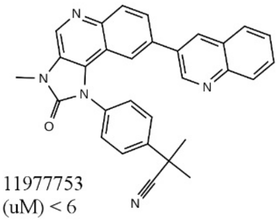<br>CID: 11977753<br>IC50 (uM) < 6	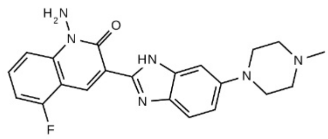<br>CID: 57336746<br>Kd (uM) = 1.5
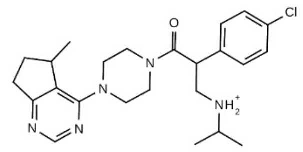<br>CID: 23649240<br>IC50 (uM) < 5		

Table S3. The Pharmacophore-Fit score values of the 25 hit compounds

Compound	Fit Score	Best fitting model
1	51.877	Model3
2	51.896	Model3
3	45.864	Model2
4	51.602	Model2
5	57.374	Model4
6	51.045	Model4
7	45.78	Model2
8	57.179	Model4
9	52.071	Model3
10	58.513	Model3
11	51.741	Model1
12	52.359	Model1
13	52.885	Model1
14	52.207	Model1
15	52.848	Model1
16	52.551	Model1
17	52.659	Model1
18	52.226	Model1
19	52.781	Model1
20	51.502	Model1
21	49.534	Model1
22	52.708	Model1
23	52.438	Model1
24	51.989	Model1
25	52.108	Model1

Table S4. Main features of cell lines utilized in the study.

CELL LINE	CANCER	FEATURES	REFERENCES
MDA-MB-231	Breast	Basal-like breast tumors (ER/PR/HER2–negative, TP53 mutations)	[1]
MCF-7	Breast	Endocrine-sensitive luminal breast tumors (ER, PR positive)	[1]
PEO1	Ovarian	High Grade Serous Ovarian cancer (TP53 mutation, germline mutation in BRCA2, impaired nucleotide excision repair capacity)	[2]; [3]; [4]
COV318	Ovarian	High Grade Serous Ovarian cancer (TP53 mutation)	[2]; [5]
NCI-H1975	Lung	Non-small cell lung cancer (EGFR mutations, p53 wild type)	[6]
NCI-H1299	Lung	Non-small cell lung cancer (N-Ras mutation, lacking p53 protein expression)	[6]
HCT-15	Colon	Microsatellite instability-high (MSI-H, ~15%), CpG island methylator phenotype, KRAS, PIK3CA and TP53 mutations	[7]; [8]
SW948	Colon	Chromosomal instability (CIN ~ 60%), KRAS, PIK3CA and TP53 mutations	[7]; [8]

References

1. Kao, J. *et al*. Molecular profiling of breast cancer cell lines defines relevant tumor models and provides a resource for cancer gene discovery. *PLoS One* **4**, e6146; 10.1371/journal.pone.0006146 (2009).

2. Beaufort, C. M. *et al*. Ovarian cancer cell line panel (OCCP): clinical importance of in vitro morphological subtypes. *PLoS One* **9**, e103988; 10.1371/journal.pone.0122284 (2014).

3. Sakai, W. *et al.* Functional restoration of BRCA2 protein by secondary BRCA2 mutations in BRCA2-mutated ovarian carcinoma. *Cancer Res*. ***69***, 6381-6386 (2009).

4. Wang, Q. E. *et al.* Differential contributory roles of nucleotide excision and homologous recombination repair for enhancing cisplatin sensitivity in human ovarian cancer cells. *Mol. Cancer* **10**, 24; 10.1186/1476-4598-10-24 (2011).

5. Domcke, S., Sinha, R., Levine, D. A., Sander, C., Schultz, N. Evaluating cell lines as tumour models by comparison of genomic profiles. *Nat. Commun*. **4**, 2126; 10.1038/ncomms3126 (2013).

6. Kellar, A., Egan, C., Morris, D. Preclinical murine models for lung cancer: clinical trial applications. *BioMed. Res. Int*. **2015**, 621324; 10.1155/2015/621324 (2015).

7. Ahmed, D. *et al*. Epigenetic and genetic features of 24 colon cancer cell lines. *Oncogenesis* **2**, e71; 10.1038/oncsis.2013.35 (2013).

8. Mouradov, D. *et al*. Colorectal cancer cell lines are representative models of the main molecular subtypes of primary cancer. *Cancer Res.* **74**, 3238-3247 (2014).
